# Evaluating short-term patient outcomes after HIV care interventions in a low resource setting: preparing for an HIV vaccine trial site in Bamako, Mali

**DOI:** 10.1186/1742-4690-9-S2-P128

**Published:** 2012-09-13

**Authors:** Z Koty, A Bicki, Y Koné, L Diarra, K Tounkara, F Siby Diallo, L Levitz, B Aboubacar, M Rochas, S Dao, A De Groot

**Affiliations:** 1 GAIA Vaccine Foundation, Bamako, Mali; 2 ASACOMSI, Mékin-Sikoro, Mali; 3 Ministry of Health, Bamako, Mali; 4 Department of Infectious Diseases, University of Bamako, Bamako, Mali; 5 GAIA Vaccine Foundation and the University of Rhode Island, Providence, RI, USA

## Background

HIV treatment in Mali is constrained by limited access to HIV experts. In 2009, GAIA Vaccine Foundation’s “Hope Center Clinic” (HCC), a community health center in Mékin-Sikoro, became one of the first “front-line” clinics to offer HIV treatment in Mali. To assess our HIV prevention strategies and prepare for an eventual HIV vaccine trial, we performed a retrospective study of ten clinical parameters in patient charts.

## Methods

The charts of 54 patients receiving antiretroviral therapy (ART) at HCC between 04/2009-09/2011 (30 months) were reviewed. Information on the presence of opportunistic infections (OIs), weight changes, CD4 counts, BMI, viral load (VL), CD4 T-cell counts, hemoglobin (Hb), alanine aminotransferase, leukocyte counts, and platelet counts were tabulated and analyzed in Excel.

## Results

The mean age of the subjects in the study was 33; 85% were women, of whom 57% received mother-to-child transmission prevention at HCC. 93% had HIV-1; 33% were WHO Stage I, 11% Stage 2, 24% Stage 3, and 4% Stage 4. 48 (89%) patients improved in at least one parameter. 35 (65%) patients gained weight. 28 (70%) patients had increased CD4 counts (74% of patients had two counts recorded). 13 (59%) patients had decreased VL (41% of patients had two VL recorded). OIs were common among subjects (61%) prior to clinical intervention, but decreased significantly by months 7-15 to affect only 17% of subjects.

## Conclusion

These data affirm that village-level HIV care is both feasible and associated with positive patient outcomes. Providing care allowed GAIA VF to reinforce the rapport between clinic staff and community members and assess clinical interventions for HIV-positive patients. Importantly, this develops strategies for treatment distribution and adherence monitoring, patient follow-up and retention, and study implementation and analysis that lay the groundwork for the development of a Phase I-III HIV vaccine trial site in this region of Mali.

